# Elevated plasma eosinophil-derived neurotoxin is associated with echocardiographic pulmonary vascular involvement in stable COPD

**DOI:** 10.3389/fmed.2026.1966419

**Published:** 2026-09-23

**Authors:** Xiaochen Hu, Fushi Dong, Qi Song, Lu Liu, Shihuan Yu

**Affiliations:** Department of Respiratory Medicine, Qunli Branch, The First Affiliated Hospital of Harbin Medical University, Harbin, Heilongjiang, China

**Keywords:** chronic obstructive pulmonary disease, diffusing capacity, echocardiographic pulmonary vascular involvement, echocardiography, eosinophil-derived neurotoxin, pulmonary circulation

## Abstract

**Background:**

Pulmonary vascular involvement is an important dimension of chronic obstructive pulmonary disease (COPD) heterogeneity, but its biological features may not be fully represented by airflow limitation, gas-transfer impairment, or resting hypoxemia. eosinophil-derived neurotoxin (EDN) is a circulating eosinophil-derived protein whose relationship with pulmonary vascular involvement in stable COPD is unknown.

**Methods:**

This single-center observational cohort study included 53 clinically stable participants with COPD and 10 healthy controls. COPD participants were classified by echocardiographic pulmonary vascular involvement (*n* = 10) or its absence (*n* = 43). Right-heart catheterization was not used for group assignment. EDN was measured in EDTA plasma by enzyme-linked immunosorbent assay. The primary analysis estimated the adjusted EDN difference between COPD groups using ordinary least-squares regression with HC3 robust standard errors. Covariates were age, sex, forced expiratory volume in 1 s, diffusing capacity for carbon monoxide, and room-air arterial oxygen tension. Secondary models examined adjusted associations with tricuspid regurgitation (TR) pressure gradient and echocardiographic pulmonary artery-to-aorta ratio (PA/Ao).

**Results:**

Median plasma EDN was 181.58 ng/mL (interquartile range [IQR], 164.98–202.53) in participants with echocardiographic pulmonary vascular involvement and 125.66 ng/mL (IQR, 113.14–140.54) in those without such involvement. The adjusted group difference was 48.93 ng/mL (95% CI, 30.04–67.81; *P* < 0.001). Secondary slopes were positive for TR pressure gradient (0.86 ng/mL per mmHg; 95% CI, 0.07–1.65; nominal *P* = 0.033) and PA/Ao (93.40 ng/mL per unit; 95% CI, 9.71–177.09; nominal *P* = 0.030); Holm-adjusted *P* = 0.059 for both. Leave-one-case-out estimates ranged from 44.59 to 53.37 ng/mL, with every lower 95% confidence bound above zero.

**Conclusion:**

After adjustment for measured respiratory variables, plasma EDN was higher in stable COPD with echocardiographic pulmonary vascular involvement. The corresponding patterns observed for TR pressure gradient and PA/Ao provide additional rationale for evaluating EDN in external cohorts with standardized echocardiography, paired eosinophil measurements, and invasive hemodynamic assessment.

## 1 Introduction

Chronic obstructive pulmonary disease is heterogeneous in its respiratory, cardiovascular, and inflammatory expression (1). Pulmonary vascular involvement is one clinically important dimension of this heterogeneity. In some patients, pulmonary vascular dysfunction and pressure elevation are more prominent than the degree of airflow obstruction would suggest, a pattern described as the pulmonary vascular phenotype of COPD (2–5). Contemporary catheterization cohorts reinforce that low diffusing capacity for carbon monoxide (DLCO), hypoxemia, and comparatively less severe airflow obstruction frequently accompany this phenotype, while its clinical course differs from end-stage parenchymal COPD (5–10). Pulmonary artery enlargement provides an additional structural signal of vascular burden in COPD (11).

Pulmonary vascular involvement in COPD is evaluated using complementary physiological and imaging measures rather than a single variable. FEV1 quantifies airflow limitation, whereas DLCO and room-air PaO2 reflect gas-transfer impairment and resting oxygenation. Disproportionately reduced DLCO can identify patients at increased pulmonary vascular risk, but these measurements do not reveal the inflammatory processes that may accompany vascular remodeling (6, 7). This gap provides a rationale for evaluating circulating biomarkers alongside established respiratory and echocardiographic measures.

Eosinophil-derived neurotoxin (EDN), also known as RNase 2, is a protein stored predominantly in eosinophil granules and measurable in circulating blood (12–16). Its analytical stability has supported its evaluation as an eosinophil-related biomarker, particularly in asthma and other eosinophilic disorders (12–14). Eosinophilic inflammation also characterizes a biologically distinct subset of COPD, although eosinophil-related signals vary across patients and lung compartments (17–19). Direct evidence on EDN in COPD remains limited and has mainly come from studies of acute exacerbations (20).

Available evidence provides a biological bridge between eosinophil-related pathways and the pulmonary circulation, although it is not specific to EDN. In a mouse model of eosinophilic inflammation, eosinophil depletion attenuated pulmonary arterial muscularization and pulmonary hypertension, whereas eosinophil granule extracts stimulated pulmonary arterial smooth-muscle-cell proliferation (21). Conversely, eosinophils were protective in another experimental model through the lipid mediators 14-HDHA and 17-HDHA (22). Human observations also appear context dependent: blood eosinophil counts were not associated with pulmonary hypertension in end-stage lung disease (23), whereas a small longitudinal study in eosinophilic granulomatosis with polyangiitis linked higher eosinophil counts to greater echocardiographic pulmonary vascular load and observed improvement in pressure-related measures during anti-IL-5/R therapy (24). Together, these findings suggest that eosinophil abundance, granule-protein release, and lipid mediators may have distinct vascular associations. Whether circulating EDN is associated with pulmonary vascular involvement in clinically stable COPD remains unknown.

We hypothesized that plasma EDN would be higher in participants with clinically stable COPD and echocardiographic pulmonary vascular involvement than in those without such involvement, after adjustment for age, sex, FEV1, DLCO, and room-air PaO2. The adjusted between-group difference in plasma EDN was designated the primary analysis. Associations of plasma EDN with TR pressure gradient and PA/Ao were evaluated as secondary, supportive analyses.

## 2 Materials and methods

### 2.1 Study design and participants

This single-center observational cohort study included participants enrolled during the initial recruitment phase of an investigator-initiated COPD biomarker study beginning in May 2025. Adults with clinically diagnosed COPD were recruited when the treating team considered them clinically stable. At study sampling, clinical stability required no acute exacerbation requiring escalation of therapy. The study protocol did not specify a fixed exacerbation-free or acute-respiratory-infection-free interval before blood sampling. Information on recent respiratory infections, corticosteroid or antibiotic exposure, and changes in maintenance treatment was not available as standardized participant-level variables in the analysis dataset. Blood sampling, arterial blood-gas analysis, pulmonary-function assessment, and echocardiographic data collection represented the baseline study evaluation. Participants were breathing room air at blood sampling and arterial blood-gas assessment.

COPD eligibility followed contemporaneous Global Initiative for Chronic Obstructive Lung Disease diagnostic criteria (1). The study excluded major alternative respiratory disorders, active malignant disease, cardiovascular conditions expected to dominate the clinical presentation, and baseline left ventricular diastolic dysfunction. For the echocardiographic pulmonary vascular involvement group, COPD was accompanied by a clinical echocardiography report showing elevated ePASP according to the source clinical classification. Pulmonary hypertension attributable to congenital heart disease, connective-tissue disease, or another identified primary cause was excluded. Ten healthy participants provided a contextual reference distribution for plasma EDN. The inferential models were restricted to the 53 participants with COPD.

The protocol was approved by the Ethics Committee of the First Affiliated Hospital of Harbin Medical University. All participants provided written informed consent. Reporting follows the Strengthening the Reporting of Observational Studies in Epidemiology statement (25).

### 2.2 Echocardiographic pulmonary vascular involvement and echocardiographic measures

Group assignment followed the recorded enrollment group and required COPD plus an original clinical echocardiography report indicating elevated pulmonary artery pressure. The reported estimated pulmonary artery systolic pressure (ePASP) incorporated the TR velocity-derived pressure gradient together with a clinically estimated right-atrial-pressure component (4). The source clinical worksheet categorized reported ePASP values of 30–50, 51–70, and >70 mmHg as mild, moderate, and severe elevation, respectively. These categories describe the source clinical classification; participants were not retrospectively reclassified using a single ePASP or TR-velocity threshold. Among the 10 participants with echocardiographic pulmonary vascular involvement, ePASP ranged from 39 to 105 mmHg, TR pressure gradient from 31 to 90 mmHg, and TR velocity from 2.784 to 4.743 m/s. The estimated right-atrial-pressure value and the inferior-vena-cava diameter and inspiratory-collapse measurements used for its estimation were not separately recorded in the analysis dataset. Therefore, although ePASP was available, the specific method used to estimate right-atrial pressure could not be retrospectively verified. Right-heart size, pulmonary artery diameter, and PA/Ao were available as structural measures but were not components of the group-classification criteria. Echocardiographic measurements were obtained from routine clinical reports, without a separate blinded core-laboratory rereading. The TR pressure gradient was retained as a continuous pressure-related measure. When the original record contained the gradient but not a transcribed velocity, velocity was recovered using the simplified Bernoulli relation, TR pressure gradient=4 × velocity^2^. Pulmonary artery and aortic diameters were recorded, and PA/Ao was calculated as pulmonary artery diameter divided by aortic diameter. Right-heart catheterization was not performed for group assignment; the study group therefore represents echocardiographic pulmonary vascular involvement rather than hemodynamically confirmed pulmonary hypertension.

### 2.3 Respiratory and clinical measurements

Post-bronchodilator FEV1 and DLCO were expressed as percentages of predicted values. The FEV1/DLCO ratio was retained as a descriptive measure of disproportionate gas-transfer impairment. Arterial PaO2, arterial carbon-dioxide tension (PaCO2), and pH were measured while participants breathed room air. Age, sex, body-mass index, smoking status, plasma albumin, and B-type natriuretic peptide were extracted from the baseline clinical evaluation. Sex was modeled as a binary covariate using the recorded biological sex. Paired peripheral blood eosinophil counts at the study sampling visit were not available in the analysis dataset. Recent respiratory infections, systemic or inhaled corticosteroid exposure, antibiotic use, other anti-inflammatory therapy, renal-function measures, documented asthma overlap or other eosinophilic disorders were not available as standardized participant-level variables in the analysis dataset. Although the eligibility criteria excluded major alternative respiratory disorders, the available analytic data did not permit separate identification or adjustment for asthma overlap or other eosinophilic disease.

### 2.4 Plasma EDN measurement

Venous blood was collected into EDTA anticoagulant tubes, centrifuged, and the separated plasma was stored at −80 °C until analysis. Plasma EDN was quantified using a commercial human EDN sandwich enzyme-linked immunosorbent assay. Samples were diluted fivefold before assay. Concentrations were calculated from the calibration curve and multiplied by the dilution factor; all curve-equivalent sample concentrations were within the 10–160 ng/mL calibration interval. Final concentrations are reported in ng/mL.

The archived assay output contained one reported well for each healthy control and each COPD participant without echocardiographic pulmonary vascular involvement. Samples from participants with echocardiographic pulmonary vascular involvement were represented by three technical wells, and their mean concentration was used in the primary analysis. The median technical-well coefficient of variation in this group was 1.10% (IQR, 0.98%–1.23%). Replicate-specific models replaced the mean with each individual technical well to test the effect of replicate selection.

### 2.5 Statistical analysis

Continuous variables are summarized as median and IQR and categorical variables as number and percentage. Between-group comparisons used the Mann–Whitney U test for continuous variables and Fisher's exact test for sex. Healthy controls were included only in a secondary three-group contextual EDN comparison using the Kruskal–Wallis test, followed by pairwise Mann–Whitney U tests with Holm correction; they were not included in any multivariable model.

The primary model used plasma EDN as the continuous outcome and echocardiographic pulmonary vascular involvement status as the exposure. Ordinary least-squares regression was selected because the primary estimand was the covariate-adjusted mean difference in EDN on the original ng/mL scale; HC3 heteroscedasticity-consistent standard errors with a small-sample t reference were used to construct the 95% confidence interval. The primary covariate set comprised age, sex, FEV1 percent predicted, DLCO percent predicted, and room-air PaO2. These variables represented demographic structure, airflow limitation, gas-transfer impairment, and resting oxygenation. Unadjusted and age-and-sex-adjusted models were also fitted, and a log-EDN model tested scale sensitivity.

Two secondary models separately replaced group status with TR pressure gradient and echocardiographic PA/Ao while retaining the same covariates. Their nominal *P* values were additionally adjusted together by the Holm method. Raw Spearman correlations and partial Spearman correlations, calculated as Pearson correlations between covariate-adjusted rank residuals, provided complementary summaries. Exploratory models that included both group status and each continuous measure examined whether the total-cohort slope remained within groups. A leave-one-case-out analysis repeated the primary model after omitting each participant with pulmonary vascular involvement in turn.

The unadjusted mean and median group differences were bootstrapped with 10,000 resamples and a fixed random seed of 20260812. All tests were two-sided. Analyses used Python 3.9.21 with pandas 2.3.3, NumPy 1.24.3, SciPy 1.13.1, and statsmodels 0.14.5. All figures were generated from the participant-level analysis data in Python.

## 3 Results

### 3.1 Participant characteristics

The analysis included 53 participants with stable COPD: 10 with and 43 without echocardiographic pulmonary vascular involvement (Figure 1). Median age was 69.5 years (IQR, 61.5–76.5) among participants with echocardiographic pulmonary vascular involvement and 68.0 years (IQR, 62.5–72.0) among those without involvement; five (50.0%) and 28 (65.1%) participants, respectively, were male (Table 1).

**Figure 1:**
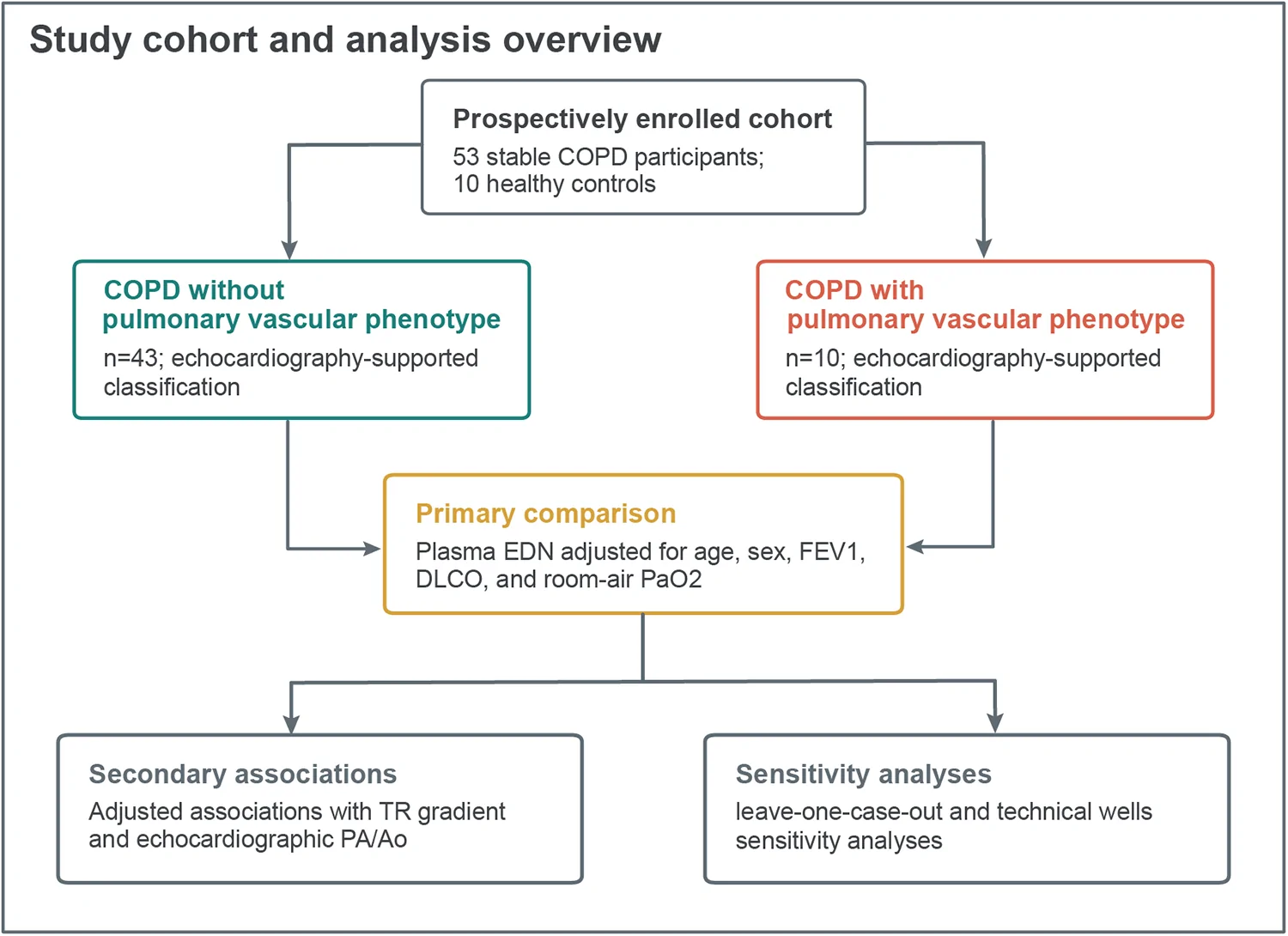
Study cohort and analysis overview. The primary inferential cohort comprised 53 clinically stable COPD participants classified by echocardiographic pulmonary vascular involvement. Ten healthy controls provided contextual EDN values. The primary model compared plasma EDN between COPD groups after adjustment for age, sex, FEV1, DLCO, and room-air PaO2. Secondary models examined TR pressure gradient and PA/Ao; sensitivity analyses used leave-one-case-out models and individual technical wells. COPD, chronic obstructive pulmonary disease; DLCO, diffusing capacity for carbon monoxide; EDN, eosinophil-derived neurotoxin; FEV1, forced expiratory volume in 1 s; PA/Ao, pulmonary artery-to-aorta ratio; PaO2, arterial oxygen tension; PV, pulmonary vascular; TR, tricuspid regurgitation.

**Table 1:** Characteristics of participants with COPD according to echocardiographic pulmonary vascular involvement .

Characteristic	Involvement-positive (*n* = 10)	Involvement-negative (*n* = 43)	*P* value
Age, years	69.50 [61.50, 76.50]	68.00 [62.50, 72.00]	0.657
Male sex, *n* (%)	5 (50.0)	28 (65.1)	0.475
BMI, kg/m2	20.30 [16.82, 23.70]	22.80 [20.54, 25.00]	0.162
FEV1, % predicted	28.15 [19.07, 59.27]	43.50 [31.40, 59.25]	0.312
DLCO, % predicted	46.35 [35.92, 53.72]	70.40 [58.55, 81.25]	<0.001
FEV1/DLCO ratio	0.72 [0.52, 1.51]	0.65 [0.49, 0.84]	0.251
PaO2 on room air, mmHg	57.90 [51.40, 59.70]	76.00 [68.30, 80.75]	<0.001
PaCO2 on room air, mmHg	46.70 [40.88, 54.98]	40.00 [38.50, 43.35]	0.097
TR gradient, mmHg	37.50 [36.00, 49.25]	24.00 [20.00, 27.00]	<0.001
TR velocity, m/s	3.06 [3.01, 3.50]	2.45 [2.24, 2.60]	<0.001
Right-heart dimension, mm	31.50 [30.00, 34.75]	32.00 [30.00, 32.00]	0.452
Pulmonary artery diameter, mm	24.50 [23.00, 27.25]	23.00 [22.00, 24.00]	0.113
Echocardiographic PA/Ao	0.94 [0.86, 1.00]	0.79 [0.74, 0.84]	<0.001
BNP (available *n* = 10/38)	331.15 [271.90, 482.30]	67.80 [41.67, 87.20]	<0.001
Albumin, g/L (available *n* = 10/41)	36.15 [34.53, 40.60]	39.30 [37.60, 42.27]	0.090
Plasma EDN, ng/mL	181.58 [164.98, 202.53]	125.66 [113.14, 140.54]	<0.001

Data are median [IQR] unless otherwise indicated. *P* values are from Mann–Whitney U tests or Fisher's exact test. Values reported below the BNP assay limit were excluded from numerical summaries; row-specific available sample sizes are shown for BNP and albumin. BNP, B-type natriuretic peptide; COPD, chronic obstructive pulmonary disease; DLCO, diffusing capacity for carbon monoxide; EDN, eosinophil-derived neurotoxin; FEV1, forced expiratory volume in 1 s; PA/Ao, pulmonary artery-to-aorta ratio; PaCO2, arterial carbon-dioxide tension; PaO2, arterial oxygen tension; PV, pulmonary vascular; TR, tricuspid regurgitation.

Participants with echocardiographic pulmonary vascular involvement had lower gas-transfer capacity and room-air oxygenation, together with stronger echocardiographic and cardiac signals. Median DLCO was 46.35% predicted (IQR, 35.92–53.72) versus 70.40% predicted (IQR, 58.55–81.25), and median PaO2 was 57.90 mmHg (IQR, 51.40–59.70) versus 76.00 mmHg (IQR, 68.30–80.75). Median TR gradient was 37.50 mmHg (IQR, 36.00–49.25) versus 24.00 mmHg (IQR, 20.00–27.00), while median PA/Ao was 0.94 (IQR, 0.86–1.00) versus 0.79 (IQR, 0.74–0.84). FEV1 was lower among participants with echocardiographic pulmonary vascular involvement, but its between-group difference was smaller than the differences in DLCO and PaO2.

### 3.2 EDN distribution and the primary between-group association

Plasma EDN showed an ordered distribution across the three contextual groups (Figure 2A). Mean EDN was 89.65 ng/mL in healthy controls, 126.31 ng/mL in COPD without echocardiographic pulmonary vascular involvement, and 181.90 ng/mL in COPD with echocardiographic pulmonary vascular involvement. The global three-group comparison was significant (Kruskal–Wallis *P* < 0.001); Holm-adjusted pairwise *P* values were < 0.001 for each comparison.   

**Figure 2:**
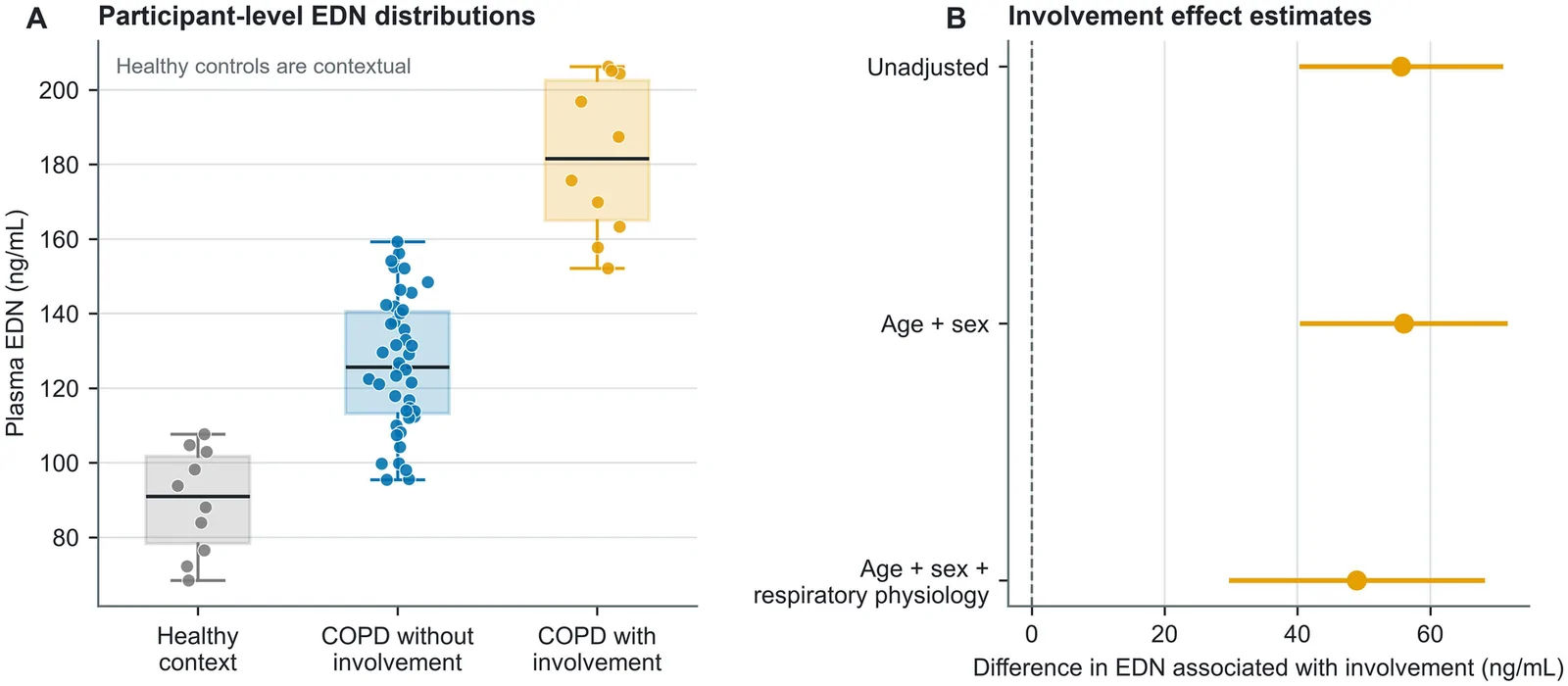
Plasma EDN distributions and echocardiographic pulmonary vascular involvement effect estimates. **(A)** Participant-level EDN distributions in healthy contextual controls, COPD without echocardiographic pulmonary vascular involvement, and COPD with echocardiographic pulmonary vascular involvement. Boxes show median and interquartile range; whiskers extend to 1.5 times the interquartile range; points represent participants. The global comparison used the Kruskal–Wallis test (*P* < 0.001), followed by two-sided pairwise Mann–Whitney U tests with Holm correction (all Holm-adjusted *P* < 0.001). **(B)** Estimated EDN difference associated with echocardiographic pulmonary vascular involvement before adjustment, after adjustment for age and sex, and after additional adjustment for FEV1, DLCO, and room-air PaO2. Points are ordinary least-squares estimates and horizontal lines are HC3 robust 95% confidence intervals. *P* values were obtained from two-sided t tests of the involvement coefficient using HC3 robust standard errors; *P* < 0.001 for each of the three models. .

Within COPD, median EDN was 181.58 ng/mL (IQR, 164.98–202.53) among participants with echocardiographic pulmonary vascular involvement and 125.66 ng/mL (IQR, 113.14–140.54) among those without involvement (Mann–Whitney *P* < 0.001). The rank-biserial correlation was 0.977. The bootstrapped mean difference was 55.59 ng/mL (95% CI, 42.17–68.73), and the bootstrapped median difference was 55.91 ng/mL (95% CI, 34.42–79.45).

The estimated difference was 55.59 ng/mL (95% CI, 40.62–70.56) before adjustment and 55.99 ng/mL (95% CI, 40.72–71.26) after adjustment for age and sex. In the primary model additionally adjusted for FEV1, DLCO, and room-air PaO2, pulmonary vascular involvement remained associated with 48.93 ng/mL higher EDN (95% CI, 30.04–67.81; *P* < 0.001; Figure 2B and Table 2). The log-EDN model yielded an adjusted geometric-mean ratio of 1.38 (95% CI, 1.21–1.56; *P* < 0.001).

**Table 2:** Association between echocardiographic pulmonary vascular involvement and plasma EDN .

Model	*n*	Estimate	95% CI	*P* value	R2
Unadjusted	53	55.59	40.62 to 70.56	<0.001	0.593
Age and sex adjusted	53	55.99	40.72 to 71.26	<0.001	0.599
Primary respiratory-adjusted	53	48.93	30.04 to 67.81	<0.001	0.624
Log-EDN sensitivity	53	1.38 ratio	1.21 to 1.56	<0.001	0.563

Estimates are group-associated EDN differences in ng/mL except for the log-EDN model, which reports the adjusted ratio of geometric means. The primary model adjusts for age, sex, FEV1 percent predicted, DLCO percent predicted, and room-air PaO2. Confidence intervals and *P* values use HC3 robust standard errors with a small-sample t reference.

### 3.3 Associations with continuous echocardiographic measures

Plasma EDN was positively associated with both continuous echocardiographic measures in the total cohort (Figure 3 and Table 3). The raw Spearman correlation between EDN and TR pressure gradient was 0.429 (*P* = 0.001). After adjustment for age, sex, FEV1, DLCO, and room-air PaO2, each 1-mmHg increase in TR pressure gradient was associated with 0.86 ng/mL higher EDN (95% CI, 0.07–1.65; nominal *P* = 0.033). The partial Spearman correlation was 0.294 (nominal *P* = 0.043).

**Figure 3:**
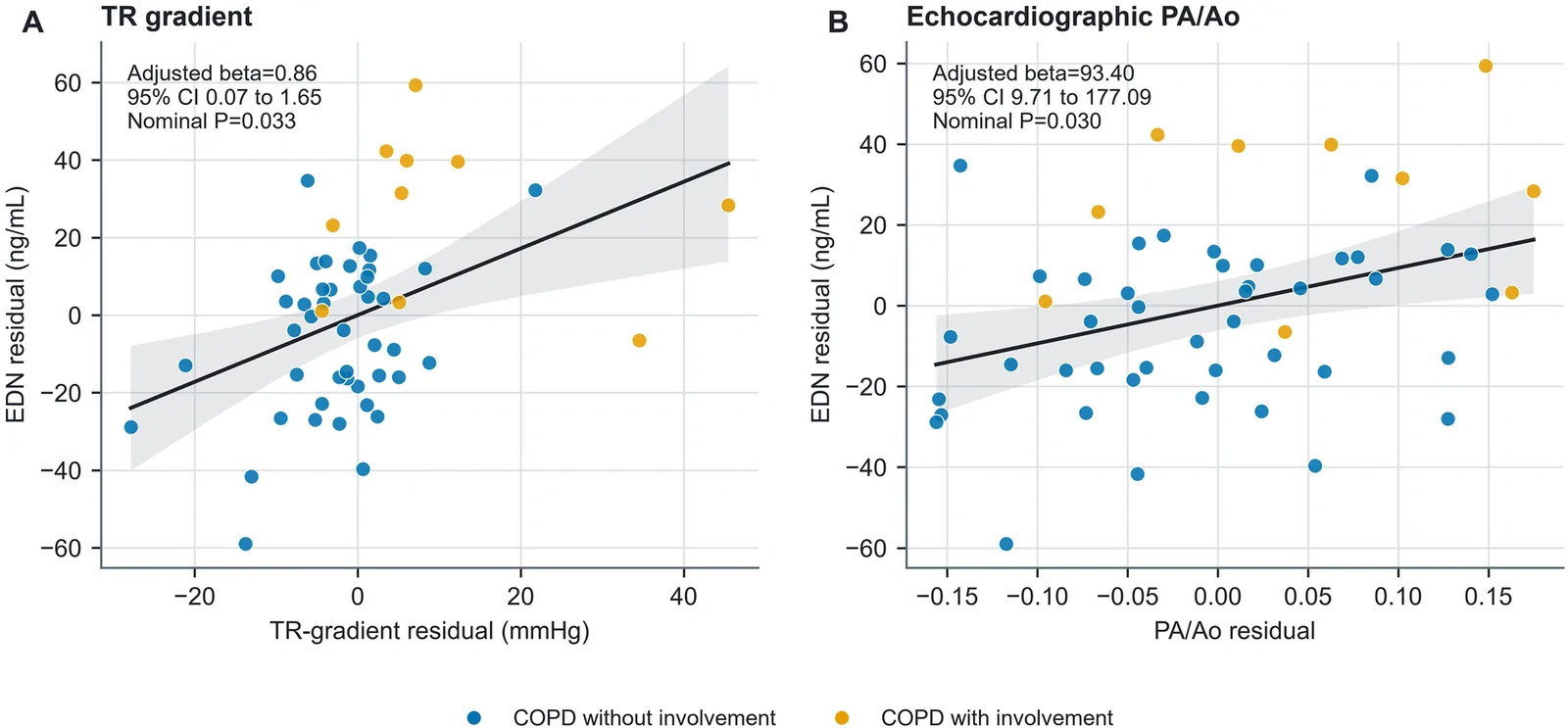
Adjusted associations of plasma EDN with continuous echocardiographic measures. Partial-regression plots show residualized EDN against residualized **(A)** TR pressure gradient and **(B)** echocardiographic PA/Ao after adjustment for age, sex, FEV1, DLCO, and room-air PaO2. Lines are fitted total-cohort associations and shaded bands are 95% confidence intervals. Panel A: adjusted coefficient=0.86 ng/mL per mmHg (95% CI, 0.07–1.65; nominal *P* = 0.033), partial Spearman rho=0.294 (nominal *P* = 0.043). Panel B: adjusted coefficient=93.40 ng/mL per unit PA/Ao (95% CI, 9.71–177.09; nominal *P* = 0.030), partial Spearman rho=0.313 (nominal *P* = 0.031). Holm-adjusted *P* = 0.059 for both regression coefficients and *P* = 0.061 for both partial correlations. When echocardiographic pulmonary vascular involvement status was added to the models, the TR-gradient coefficient was 0.03 ng/mL per mmHg (95% CI, −0.81 to 0.87; *P* = 0.938), and the PA/Ao coefficient was 40.42 ng/mL per unit (95% CI, −26.63 to 107.47; *P* = 0.231). These continuous-measure analyses are supportive descriptions of the primary group comparison rather than independent evidence. Colors indicate echocardiographic pulmonary vascular involvement status.   .

**Table 3:** Adjusted associations of plasma EDN with continuous echocardiographic measures.

Measure	*n*	Adjusted beta	95% CI	*P* value	Partial rho	Partial *P*
TR gradient	53	0.86	0.07 to 1.65	0.033	0.294	0.043
Echocardiographic PA/Ao	53	93.40	9.71 to 177.09	0.030	0.313	0.031

Models adjust for age, sex, FEV1 percent predicted, DLCO percent predicted, and room-air PaO2. Confidence intervals and nominal *P* values use HC3 robust standard errors with a small-sample t reference. Holm-adjusted *P* = 0.059 for both secondary regression models. The TR-gradient beta is in ng/mL per mmHg. The PA/Ao beta is in ng/mL per unit (equivalent to 9.34 ng/mL per 0.10). Partial rho is the Pearson correlation between rank residuals after covariate adjustment; Holm-adjusted partial-correlation *P* = 0.061 for both measures.

The raw Spearman correlation between EDN and echocardiographic PA/Ao was 0.512 (*P* < 0.001). In the adjusted model, a one-unit increase in PA/Ao was associated with 93.40 ng/mL higher EDN (95% CI, 9.71–177.09; nominal *P* = 0.030), equivalent to 9.34 ng/mL per 0.10 higher PA/Ao. The partial Spearman correlation was 0.313 (nominal *P* = 0.031). The secondary regression slopes and partial correlations were directionally positive but did not meet the conventional 0.05 threshold after Holm correction (adjusted *P* = 0.059 for both regression slopes and adjusted *P* = 0.061 for both partial correlations). When echocardiographic pulmonary vascular involvement status was added to the continuous models, the TR-gradient slope attenuated to 0.03 ng/mL per mmHg (95% CI, −0.81 to 0.87; *P* = 0.938), and the PA/Ao slope attenuated to 40.42 ng/mL per unit (95% CI, −26.63 to 107.47; *P* = 0.231). These continuous-measure analyses are therefore interpreted as supportive descriptions of the primary group comparison rather than independent evidence.           

### 3.4 Influence and technical-well sensitivity analyses

No single participant with echocardiographic pulmonary vascular involvement accounted for the primary adjusted estimate. Across the 10 leave-one-case-out models, adjusted group differences ranged from 44.59 to 53.37 ng/mL; lower 95% confidence bounds ranged from 26.44 to 35.91 ng/mL (Figure 4). Replacing the triplicate mean with technical well 1, 2, or 3 produced adjusted differences of 48.84, 49.71, and 48.23 ng/mL, respectively (Supplementary Figure 2).

**Figure 4:**
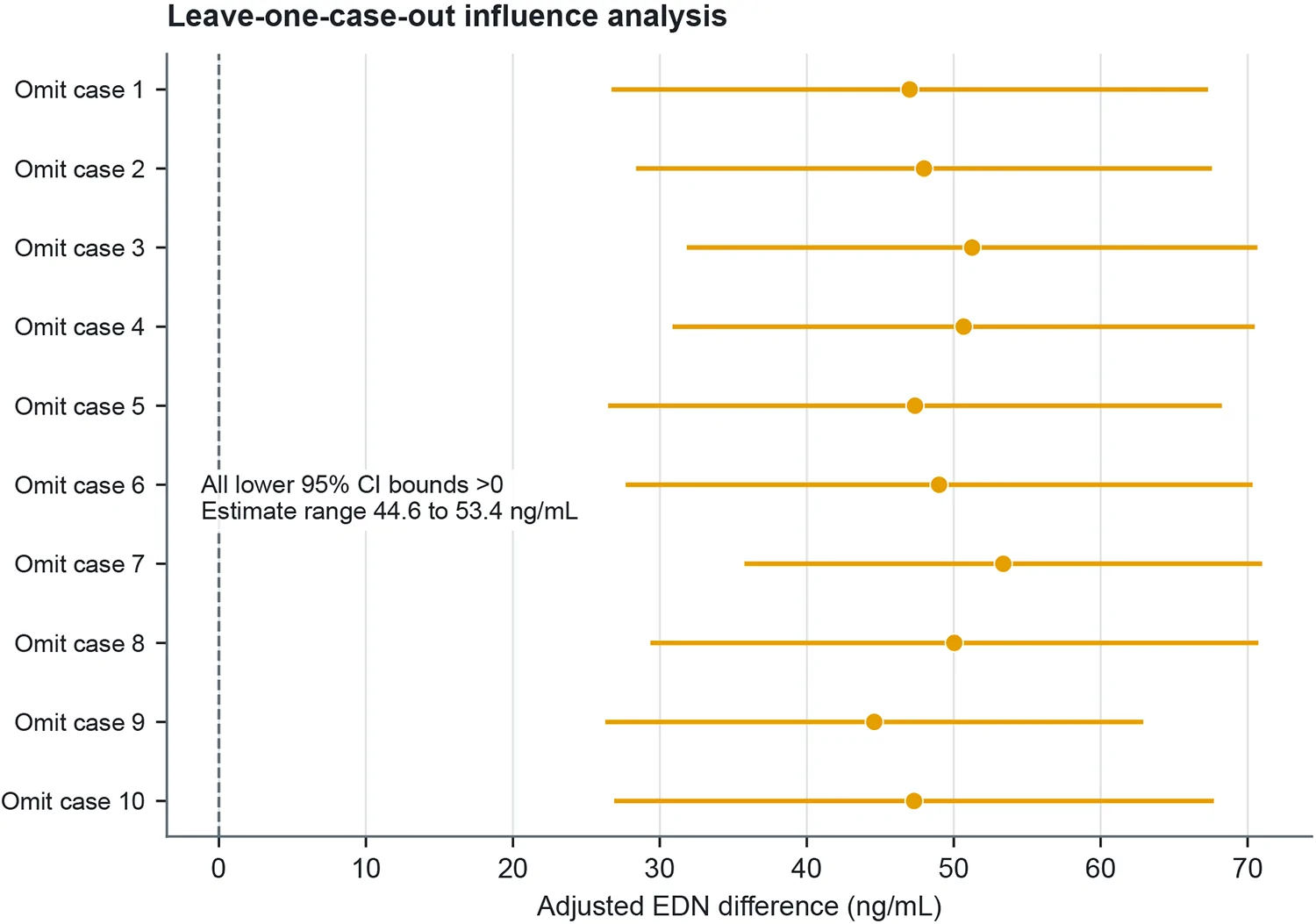
Leave–OnE,casE–out influence analysis. The primary adjusted model was repeated after omitting each participant with echocardiographic pulmonary vascular involvement in turn. Points are adjusted EDN differences and horizontal lines are 95% confidence intervals.

## 4 Discussion

In this single-center observational cohort of clinically stable COPD, plasma EDN was higher in participants with echocardiographic pulmonary vascular involvement after adjustment for age, sex, FEV1, DLCO, and room-air PaO2. EDN was also associated with TR pressure gradient and PA/Ao. The adjusted group difference changed little in leave-one-case-out analyses, indicating that no single participant with echocardiographic pulmonary vascular involvement accounted for the result.

Low DLCO and hypoxemia are characteristic of pulmonary vascular-predominant COPD and help identify patients whose vascular burden is greater than airflow obstruction alone would suggest (2, 5–7). The present cohort reproduced this pattern: DLCO and PaO2 differed more markedly between groups than FEV1. The EDN difference persisted when FEV1, DLCO, and room-air PaO2 entered the same model. This result indicates an association conditional on the measured respiratory variables; it does not establish pulmonary vascular specificity or exclude residual confounding by disease severity, inflammation, treatment exposure, or comorbidity.

The secondary echocardiographic analyses clarify the structure of the main comparison. TR pressure gradient is a pressure-related measure closely related to the ePASP documented in the source clinical reports, whereas PA/Ao expresses pulmonary arterial size relative to the aorta; pulmonary artery enlargement has established clinical relevance in COPD (11). Both total-cohort slopes were positive, but their Holm-adjusted *P* values were 0.059, and the slopes attenuated when group status entered the models. The continuous analyses therefore show how the primary group difference is distributed across related pressure and structural measurements. They support the internal consistency of the echocardiographic profile while keeping the adjusted categorical comparison at the center of the inference.

EDN provides a specific link to eosinophil biology. It is RNase 2, a protein stored predominantly in eosinophil granules and measurable in circulating samples (12–16). Eosinophil-related biology in COPD varies across patients and tissue compartments. Airway transcriptomic studies connect eosinophilia with a type 2 program and corticosteroid response, while tissue studies show spatially organized eosinophil- and basophil-rich microenvironments (17–19). Previous COPD studies of EDN have largely examined acute exacerbation and thromboembolic settings (20). The current finding extends that literature to a cross-sectional association with pulmonary vascular involvement during clinical stability.

The biological meaning of this association is likely to depend on which eosinophil-related process EDN represents. Eosinophil depletion reduced pulmonary arterial muscularization in one mouse model, and eosinophil granule extracts stimulated pulmonary arterial smooth-muscle proliferation (21). In another experimental model, the eosinophil-derived lipid mediators 14-HDHA and 17-HDHA were protective (22). Blood eosinophil counts were similar in patients with and without pulmonary hypertension in a cohort with end-stage lung disease (23). Recent longitudinal evidence in eosinophilic granulomatosis with polyangiitis linked higher peripheral eosinophil counts to greater echocardiographic pulmonary vascular load and impaired RV–PA coupling, with improvement in pressure-related measures following anti-IL-5/R therapy (24). Although EGPA and COPD are distinct disease settings, these findings provide complementary human context for a potential relationship between eosinophil-related biology and pulmonary vascular alterations. These observations concern different products and contexts: cell abundance, granule proteins, and lipid mediators need not vary together or have the same vascular effects. Because paired eosinophil counts were unavailable, the present data cannot determine whether plasma EDN reflects eosinophil abundance, granule release, altered clearance, or a broader inflammatory state; EDN should therefore be interpreted as an eosinophil-derived protein rather than a surrogate for eosinophil count or activation.

The immediate clinical value of the finding is a testable biomarker hypothesis. Pulmonary vascular assessment in COPD already integrates symptoms, lung function, gas exchange, imaging, echocardiography, and, in selected patients, right heart catheterization (3–7). A decisive next study would measure EDN and blood eosinophils in a larger stable-COPD cohort with standardized, blinded echocardiography and invasive hemodynamics. That design could determine whether EDN adds information to routine respiratory measurements and whether its concentration follows pulmonary vascular resistance or pressure severity.

Only 10 of the 53 COPD participants had echocardiographic pulmonary vascular involvement. Because the primary model included group status and five covariates, overfitting and collinearity remain possible, and the adjusted estimate may be imprecise or sensitive to group composition. The similarity of the unadjusted, age-and-sex-adjusted, fully adjusted, log-scale, and leave-one-case-out estimates provides an internal stability check, but these analyses do not eliminate small-sample uncertainty or substitute for external validation.

Several design features set the precision of the current estimate. The result concerns noninvasive echocardiographic pulmonary vascular involvement, and the single-center involvement-positive group contained 10 participants. Group assignment followed the recorded enrollment group supported by the original clinical echocardiography report rather than a retrospectively applied single ePASP or TR-velocity threshold. The estimated right-atrial-pressure value and the inferior-vena-cava measurements used for its estimation were not separately recorded, and no separate blinded core-laboratory rereading was performed. Right-heart size, pulmonary artery diameter, and PA/Ao were available but were not components of the group-classification criteria. Because right-heart catheterization was not performed, the findings should be interpreted as an association with echocardiographic pulmonary vascular involvement rather than hemodynamically confirmed pulmonary hypertension. The cross-sectional sample characterizes EDN at a single clinically stable assessment. Clinical stability was determined by the treating team at sampling, without a prespecified exacerbation-free or acute-respiratory-infection-free interval. Information on recent respiratory infections, systemic or inhaled corticosteroid exposure, antibiotic use, other anti-inflammatory therapy, changes in maintenance treatment, renal-function measures, documented asthma overlap or other eosinophilic disorders, and paired peripheral blood eosinophil counts was not available as standardized participant-level variables. These unmeasured factors may have contributed residual variability and biological confounding. The observed association should therefore be interpreted as a biomarker signal rather than evidence of a specific eosinophil-driven vascular mechanism. Assay records contained three technical wells for the involvement-positive group and one reported well for the other groups. The nearly identical estimates obtained from each involvement-positive well show that the result was insensitive to well selection; balanced and blinded assay runs are the appropriate setting for testing group-linked run effects and the reproducibility of absolute EDN concentrations. The concentration scale reported here therefore applies to EDTA plasma measured with this assay, whereas the adjusted association is the main target for independent replication.

## 5 Conclusion

Plasma EDN was associated with echocardiographic pulmonary vascular involvement in stable COPD after adjustment for FEV1, DLCO, and room-air PaO2. Independent studies using balanced assays, paired eosinophil measures, standardized echocardiography, and invasive hemodynamics can test the reproducibility and biological meaning of this association.

## Data Availability

The datasets presented in this article are not readily available because the deidentified participant-level data are subject to institutional ethics and privacy requirements and may be made available by the corresponding author upon reasonable request and institutional approval. Requests to access the datasets should be directed to Shihuan Yu, yushihuan2000@126.com.

## References

[1] Agustí A Celli BR Criner GJ Halpin D Anzueto A Barnes P. Global initiative for chronic obstructive lung disease 2023 report: gOLD executive summary. Eur Respir J. (2023) 61:2300239. 10.1183/13993003.00239-2023 PMC10066569 36858443

[2] Kovacs G Agusti A Barberà JA Celli B Criner G Humbert M. Pulmonary vascular involvement in chronic obstructive pulmonary disease. Is there a pulmonary vascular phenotype? Am J Respir Crit Care Med. (2018) 198:1000–11. 10.1164/rccm.201801-0095PP 29746142

[3] Shlobin OA Adir Y Barbera JA Cottin V Harari S Jutant E-M. Pulmonary hypertension associated with lung diseases. Eur Respir J. (2024) 64:2401200. 10.1183/13993003.01200-2024 39209469 PMC11525344

[4] Humbert M Kovacs G Hoeper MM Badagliacca R Berger RMF Brida M. 2022 ESC/ERS guidelines for the diagnosis and treatment of pulmonary hypertension. Eur Respir J. (2023) 61:2200879. 10.1183/13993003.00879-2022 36028254

[5] Steger M Canuet M Martin G Labani A Schwartz JC Enache I. Pulmonary hypertension associated with COPD: a phenotype analysis. ERJ Open Res. (2025) 11:00716–2024. 10.1183/23120541.00716-2024 40129550 PMC11931555

[6] Balasubramanian A Hemnes AR Brittain EL Annis J Fawzy A Putcha N. Disproportionate impairment in diffusing capacity predicts pulmonary hypertension with an elevated pulmonary vascular resistance in COPD. Thorax. (2026) 81:524–32. 10.1136/thorax-2025-223921 41309285 PMC13098466

[7] Nathan SD Piccari L Abman SH Balasubramanian A Girgis RE Kovacs G. Significance of pulmonary vascular dysfunction in chronic obstructive pulmonary disease. Pulm Circ. (2025) 15:e70144. 10.1002/pul2.70144 40832443 PMC12360464

[8] Steger M Canuet M Enache I Goetsch T Labani A Meyer L. Survival and response to pulmonary vasodilator therapies in patients with chronic obstructive pulmonary disease and pulmonary vascular phenotype. Respir Med. (2024) 225:107585. 10.1016/j.rmed.2024.107585 38479707

[9] Hurdman J Condliffe R Elliot CA Swift A Rajaram S Davies C. Pulmonary hypertension in COPD: results from the ASPIRE registry. Eur Respir J. (2013) 41:1292–301. 10.1183/09031936.00079512 23018917

[10] Dauriat G Reynaud-Gaubert M Cottin V Lamia B Montani D Canuet M. Severe pulmonary hypertension associated with chronic obstructive pulmonary disease: a prospective French multicenter cohort. J Heart Lung Transplant. (2021) 40:1009–18. 10.1016/j.healun.2021.04.021 34218966

[11] Wells JM Washko GR Han MLK Abbas N Nath H Mamary AJ. Pulmonary arterial enlargement and acute exacerbations of COPD. N Engl J Med. (2012) 367:913–21. 10.1056/NEJMoa1203830 22938715 PMC3690810

[12] Rutten B Young S Rhedin M Olsson M Kurian N Syed F. Eosinophil-derived neurotoxin: a biologically and analytically attractive asthma biomarker. PLoS One. (2021) 16:e0246627. 10.1371/journal.pone.0246627 33566823 PMC7875349

[13] Karsten C Stier T Wood-Wentz C Smith C Yang YK Snyder M. Evaluation of an automated assay for eosinophil-derived neurotoxin in serum. Clin Biochem. (2025) 136:110890. 10.1016/j.clinbiochem.2025.110890 39864608

[14] Özuygur Ermis SS Malmhäll C Borres MP Movérare R Lisik D Abohalaka R. Serum and nasal lavage fluid eosinophil-derived neurotoxin levels and their determinants in adults. J Allergy Clin Immunol Glob. (2025) 4:100510. 10.1016/j.jacig.2025.100510 PMC12270069 40677614

[15] Yang D Chen Q Su SB Zhang P Kurosaka K Caspi RR. Eosinophil-derived neurotoxin acts as an alarmin to activate the TLR2–MyD88 signal pathway in dendritic cells and enhances Th2 immune responses. J Exp Med. (2008) 205:79–90. 10.1084/jem.20062027 18195069 PMC2234357

[16] Rosenberg H. Eosinophil-derived neurotoxin/RNase 2: connecting the past, the present and the future. CPB. (2008) 9:135–40. 10.2174/138920108784567236 PMC2680432 18673278

[17] Lee Y-L Heriyanto DS Yuliani FS Laiman V Choridah L Lee K-Y. Eosinophilic inflammation: a key player in COPD pathogenesis and progression. Ann Med. (2024) 56:2408466. 10.1080/07853890.2024.2408466 39624959 PMC11459840

[18] Leung C Park HY Li X Koelwyn GJ Tuong J Vahedi SM. Transcriptomic profiling of the airway epithelium in COPD links airway eosinophilia to type 2 inflammation and corticosteroid response. Eur Respir J. (2025) 65:2401875. 10.1183/13993003.01875-2024 39978857

[19] Jogdand P Siddhuraj P Mori M Sanden C Jönsson J Walls AF. Eosinophils, basophils and type 2 immune microenvironments in COPD-affected lung tissue. Eur Respir J. (2020) 55:1900110. 10.1183/13993003.00110-2019 32060064 PMC7236868

[20] Yang Q-F Lu T-T Shu C-M Feng L-F Chang H-T Ji Q-Y. Eosinophilic biomarkers for detection of acute exacerbation of chronic obstructive pulmonary disease with or without pulmonary embolism. Exp Ther Med. (2017) 14:3198–206. 10.3892/etm.2017.4876 28912870 PMC5585757

[21] Weng M Baron DM Bloch KD Luster AD Lee JJ Medoff BD. Eosinophils are necessary for pulmonary arterial remodeling in a mouse model of eosinophilic inflammation-induced pulmonary hypertension. Am J Physiol Lung Cell Mol Physiol. (2011) 301:L927–36. 10.1152/ajplung.00049.2011 21908591 PMC3233831

[22] Shu T Zhang J Zhou Y Chen Z Li J Tang Q. Eosinophils protect against pulmonary hypertension through 14-HDHA and 17-HDHA. Eur Respir J. (2023) 61:2200582. 10.1183/13993003.00582-2022 36423907 PMC9978164

[23] Barnikel M Kneidinger N Gerckens M Mümmler C Lenoir A Mertsch P. Current blood eosinophilia does not predict the presence of pulmonary hypertension in patients with end-stage lung disease. JCM. (2025) 14:1120. 10.3390/jcm14041120 40004650 PMC11856528

[24] Carlucci P Marozzi MS Corvasce F Noviello S Spataro F Desantis V. Eosinophil-linked pulmonary circulation in EGPA improves under anti-IL-5/R therapy: a longitudinal study. Eur J Intern Med. (2026):107033. 10.1016/j.ejim.2026.107033 42321133

[25] Von Elm E Altman DG Egger M Pocock SJ Gøtzsche PC Vandenbroucke JP, for the STROBE Initiative. The strengthening the reporting of observational studies in epidemiology (STROBE) statement: guidelines for reporting observational studies. Ann Intern Med. (2007) 147:573–7. 10.7326/0003-4819-147-8-200710160-00010 17938396

