# Supplementary material for: Elevated plasma eosinophil-derived neurotoxin is associated with echocardiographic pulmonary vascular involvement in stable COPD
Source: Front Med (Lausanne). 2026 Sep 23;13:1966419. doi: 10.3389/fmed.2026.1966419 (PMC13642290; doi:10.3389/fmed.2026.1966419)
Supplement: Supplementary file 1 [file Supplementaryfile1.docx]

Supplementary Material

**Supplementary Table 1. Univariable Spearman correlations with plasma EDN in COPD**

| **Variable** | **n** | **Spearman rho** | **P value** |
| --- | --- | --- | --- |
| TR gradient, mmHg | 53 | 0.429 | 0.001 |
| TR velocity, m/s | 53 | 0.419 | 0.002 |
| Right-heart dimension, mm | 53 | 0.036 | 0.800 |
| Pulmonary artery diameter, mm | 53 | 0.159 | 0.256 |
| Echocardiographic PA/Ao | 53 | 0.512 | <0.001 |
| BNP | 48 | 0.360 | 0.012 |
| FEV1, % predicted | 53 | -0.243 | 0.079 |
| DLCO, % predicted | 53 | -0.518 | <0.001 |
| PaO2, mmHg | 53 | -0.423 | 0.002 |
| PaCO2, mmHg | 53 | 0.235 | 0.090 |
| Albumin, g/L | 51 | -0.155 | 0.277 |
| BMI, kg/m2 | 53 | -0.211 | 0.130 |

These correlations are descriptive and unadjusted. BNP values reported below an assay limit were omitted from the BNP correlation. Abbreviations are defined in the main manuscript.

**Supplementary Table 2. Pairwise contextual comparisons of plasma EDN**

| **Group A** | **Group B** | **U** | **Unadjusted P** | **Holm P** |
| --- | --- | --- | --- | --- |
| Healthy controls | COPD without PV phenotype | 21.0 | <0.001 | <0.001 |
| Healthy controls | COPD with PV phenotype | 0.0 | <0.001 | <0.001 |
| COPD without PV phenotype | COPD with PV phenotype | 5.0 | <0.001 | <0.001 |

Pairwise Mann-Whitney U tests followed the global Kruskal-Wallis comparison; P values were adjusted by the Holm method.

**Supplementary Table 3. Leave-one-pulmonary-vascular-case-out analysis**

| **Omitted case** | **n** | **Adjusted difference** | **95% CI** | **P value** | **R2** |
| --- | --- | --- | --- | --- | --- |
| Case 1 | 52 | 47.00 | 26.86 to 67.14 | <0.001 | 0.589 |
| Case 2 | 52 | 47.98 | 28.54 to 67.41 | <0.001 | 0.580 |
| Case 3 | 52 | 51.25 | 31.99 to 70.51 | <0.001 | 0.639 |
| Case 4 | 52 | 50.67 | 31.03 to 70.32 | <0.001 | 0.628 |
| Case 5 | 52 | 47.36 | 26.64 to 68.09 | <0.001 | 0.603 |
| Case 6 | 52 | 49.00 | 27.83 to 70.18 | <0.001 | 0.609 |
| Case 7 | 52 | 53.37 | 35.91 to 70.83 | <0.001 | 0.647 |
| Case 8 | 52 | 50.04 | 29.51 to 70.57 | <0.001 | 0.615 |
| Case 9 | 52 | 44.59 | 26.44 to 62.74 | <0.001 | 0.602 |
| Case 10 | 52 | 47.30 | 27.05 to 67.55 | <0.001 | 0.592 |

Each model omits one participant with the pulmonary vascular phenotype and retains the primary covariate set. Differences are reported in ng/mL.

**Supplementary Table 4. Technical-well sensitivity analysis**

| **Technical well** | **n** | **Adjusted difference** | **95% CI** | **P value** | **R2** |
| --- | --- | --- | --- | --- | --- |
| Replicate 1 | 53 | 48.84 | 29.77 to 67.92 | <0.001 | 0.622 |
| Replicate 2 | 53 | 49.71 | 31.14 to 68.28 | <0.001 | 0.632 |
| Replicate 3 | 53 | 48.23 | 29.10 to 67.35 | <0.001 | 0.616 |

Each model substitutes one pulmonary-vascular-phenotype technical well for the triplicate mean and retains the primary covariate set. Differences are reported in ng/mL.

Supplementary Figure 1. Univariable clinical correlation landscape. Spearman correlations between plasma EDN and available clinical, physiological, and echocardiographic variables in the COPD cohort. Coral bars indicate unadjusted P<0.05.

Supplementary Figure 2. Technical-well sensitivity. Adjusted EDN phenotype differences after replacing the pulmonary vascular phenotype triplicate mean with each technical well separately. Models adjust for age, sex, FEV1, DLCO, and room-air PaO2. Points are estimates and horizontal lines are 95% confidence intervals.
